# Distribution profile of iridoid glycosides and phenolic compounds in two *Barleria* species and their correlation with antioxidant and antibacterial activity

**DOI:** 10.3389/fpls.2022.1076871

**Published:** 2023-01-09

**Authors:** Shachi Singh, Mukesh Kumar, Seema Dwivedi, Anjali Yadav, Sarika Sharma

**Affiliations:** ^1^ Department of Botany, MMV, Banaras Hindu University, Varanasi, India; ^2^ Department of Statistics, MMV, Banaras Hindu University, Varanasi, India

**Keywords:** iridoid glycoside, metabolomic, antibacterial, antioxidant, phenolic compounds

## Abstract

**Introduction:**

*Barleria prionitis* is known for its medicinal properties from ancient times. Bioactive iridoid glycosides and phenolic compounds have been isolated from leaves of this plant. However, other parts of a medicinal plants are also important, especially roots. Therefore, it is important to screen all organs for complete chemical characterization.

**Method:**

All parts of *B. prionitis*, including leaf, root, stem and inflorescence in search of bioactive compounds, with a rapid and effective metabolomic method. X500R QTOF system with information dependent acquisition (IDA) method was used to collect high resolution accurate mass data (HRMS) on both the parent (MS signal) and their fragment ions (MS/MS signal). ESI spectra was obtained in positive ion mode from all parts of the plant. A comparative analysis of antioxidant and antibacterial activity was done and their correlation study with the identified compounds was demonstrated. Principal component analysis was performed.

**Result:**

Iridoid glycosides and phenolic compounds were identified from all parts of the showing variability in presence and abundance. Many of the compounds are reported first time in *B. prionitis*. Antioxidant and antibacterial activity was revealed in all organs, root being the most effective one. Some of the iridoid glycoside and phenolic compounds found to be positively correlated with the tested biological activity. Principal component analysis of the chemical profiles showed variability in distribution of the compounds.

**Conclusion:**

All parts of *B. prionitis* are rich source of bioactive iridoid glycosides and phenolic compounds.

## 1 Introduction

*Barleria*, a member of Acanthaceae family, is a spiny shrub and known for its medicinal properties from ancient times (Chen et al., 1998; Amoo et al., 2009). It is found in India and also distributed in different parts of Asia and Africa (Chen et al., 1998). Aerial parts of this plant has long been used in the treatment of diseases such as, toothache, whooping cough, respiratory and gastrointestinal disorders, fever, swelling, artheritis, skin disorders, jaundice etc (Amoo et al., 2009; Gangaram et al., 2022). Extracts collected from aerial parts of the plant have shown to possess antioxidant, antibacterial, antiviral, anti-inflammatory, hepatoprotective, antidiabetic, antifertility, immune modulating activity and acetylcholinesterase inhibitory activities (Chen et al., 1998; Gupta et al., 2000; Singh et al., 2003; Singh et al., 2005; Verma et al., 2005; Kosmulalage et al., 2007; Amoo et al., 2009; Dheer and Bhatnagar, 2010; Chavan et al., 2014; Sharma et al., 2014; Gangaram et al., 2022).

Iridoid glycosides and phenolics are important class of chemical compounds isolated from leaves of this plant. Iridoids are oxygenated monoterpenes that commonly occur as glycoside attached with glucose. They have been reported in several plant families and have shown a broad spectrum of biological activities (Widyowatia et al., 2010; Zhang et al., 2018a). Isolated compounds from *Barleria* species (Taneja and Tiwari, 1975; Kanchanapooma et al., 2002; Leea et al., 2016; Gangaram et al., 2022) have demonstrated to possess glutathione S-transferase inhibitory activity, acetylcholinesterase inhibitory activity, free radical scavenging, antimicrobial, anti-inflammatory, immunomodulatory and gastroprotective activities (Ata et al., 2009; Ghule and Yeole, 2012; Jaiswal et al., 2014; Zhang et al., 2018b; Sun et al., 2022). Phenolic compounds are widely distributed throughout the plant kingdom. They are involved in a variety of biological activities such as antimicrobial, anti-inflammatory, antioxidant, antidiabetic, hepatoprotective, and anticancer properties (Tanase et al., 2019; Pannakal et al., 2022). Phenolics isolated from aerial parts of *Barleria* species have shown to possess biological activities (Vertuani et al., 2011; Gangaram et al., 2022).

Although active ingredients had been identified in leaves of *Barleria species*, limited work is done to explore other parts of the plant, such as root, bark or flowers, which could also be a rich source of bioactive compounds. Therefore, the objective of this study was to identify the active compounds present in all parts of the plant and study their distribution pattern so that the full pharmacological potential of a medicinal plant could be exploited.

Hyphenated liquid chromatography and tandem mass spectrometry (LC–MS/MS) is considered to be the most suitable technique for chemical characterization of natural extracts. Although the necessity of the technique in natural product science is unquestionable, however, it is time consuming and cannot meet the demand arising due to increase in number of samples. Therefore, it is required to find a more efficient technique which can perform high-throughput analysis by overcoming the time constrains of the conventional method. Application of direct mass measurement through techniques, such as electrospray ionisation (ESI-MS) have proven useful in characterizing crude extracts, such as medicinal plants (Mauri and Pietta, 2000; Kaur and Kaur, 2016; Liu et al., 2021) essential oils (Møller et al., 2007) etc. The speed is fast, however in absence of MS/MS signals, the information may not be sufficient to identify the compounds with accuracy. If a mass spectrometry instrument can select ion currents and generate their MS1 and MS2 spectra with a single injection, the necessity of separation based techniques can be overcome. In the present experiment we have used direct mass measurement coupled with information dependent acquisition (IDA) method for generation of MS and MS/MS signals. The method was found useful in analyzing multiple batches of sample within a short interval of time.

## 2 Methods

### 2.1 Plant material and preparation of extract

*B. prionitis* and *B. cristata* were collected from the Ayurvedic Garden of Banaras Hindu University, Varanasi, India, in the month of December, 2021. The plant material was washed thoroughly under running tap water and dried in air. Different parts of the plant, such as root, stem, leaves and inflorescence were separated and kept in oven with temperature not exceeding above 50°C. Dried plant material was grinded into fine powder. About 50 g of plant powder was extracted by absolute ethanol with sonication at 45°C for 15 min with frequency 40 kHz, power 100W, the procedure was repeated thrice. The decoction was filtered using the Whatman paper, the plant material was again extracted thrice with 70% ethanol; both the filtrates were pooled together and dried at 45°C. The obtained residue was extracted with hexane, to remove non-polar compounds, the remaining residue was used for further analysis.

### 2.2 2, 2-Diphenyl-1-picrythydrazyl (DPPH) radical scavenging assay

Total free radical scavenging capacity of the extract was estimated using the stable DPPH radical, as per the method described by Singh, 2016. Test sample was prepared by dissolving 10 mg extract in 1 ml methanol and 0.004% DPPH solution was prepared by dissolving 4 mg DPPH in 100 ml methanol. A 0.5 ml of methanolic extract was diluted by adding 2 ml methanol, followed by addition of 1 ml of DPPH solution. The mixture was shaken and incubated in dark for 30 minutes at room temperature. Absorbance was measured at 517nm spectrophotometrically. Percentage of antioxidant activity was calculated using the following formula:


Radical scavenging activity %=Absorbance of Control−Absorbance of Sample/Absorbance of control∗100

Where control was 3 ml methanol with 1 ml DPPH and sample was methanolic plant extract.

Antioxidant activity of the extracts was compared with standard antioxidant (Ascorbic acid). IC_50_ value was determined from the plotted graph of scavenging activity against the different concentrations of extracts, which is defined as the total antioxidant necessary to decrease the initial DPPH radical concentration by 50%.

### 2.3 Ferric- reducing antioxidant power (FRAP) assay

Frap assay was performed according to the method of Guo et al. (2003). Frap reagent was prepared freshly by addition of 2.5 ml of 10mmol/l TPTZ (2,4,6-Tripyridy- s-triazine, sigma) in 40 mmol/l HCl and 2.5 ml of 20mmol/l FeCl3 in 25 ml of 0.3mol/l acetate buffer, pH 3.6 and kept at 37^0^C. Now a 0.5 ml of methanolic extract was taken in a test tube and diluted by 0.5 ml methanol, followed by addition of 2 ml frap reagent, the mixture was kept for 15 minutes in 37°C. Absorbance of this reaction mixture was measured at 593nm using UV/VIS spectrophotometer. Antioxidant activity was calculated by comparing with standard antioxidant (ascorbic acid).

### 2.4 Antibacterial activity

Antibacterial activity was performed by well diffusion method, according to the method decribed in literature (Singh et al., 2020), on the following test bacteria, obtained from MTCC (IMTECH, Chandigarh); *Escherichia coli, Staphylococcus aureus sub* sp. *aureus, Bacillus subtilis, Pseudomonas aeruginosa* and *Klebsiella pneumonia*. Plant extract of 10mg/ml concentration was prepared in distilled water. Nutrient agar plates were prepared and 0.5 cm wells were made on the solid media. Plates were inoculated by bacterial culture and wells were filled with 20 µl of the extract. The plates were incubated for 24h at 37°C. Antibacterial activity of the compound was determined by measuring the diameter of zone of inhibition of microbial growth. Antibiotic, Streptomycin at concentration of 1 mg/ml was used as positive control.

### 2.5 Mass spectrometry

Plant extract (10 mg/ml) of root, leaf, stem and inflorescence of *B.prionitis* and *B.cristata*, prepared in methanol, were directly analyzed by mass spectrometer. The SCIEX X500R QTOF system with the Turbo V™ source was operated in positive electrospray ionization (ESI) mode. The TOF MS scan was conducted over a range of 100-1000 m/z. Following MS parameters were selected; ion source gas one 60 psi, ion source gas two 60 psi, curtain gas 40 psi, source temperature 400-500°C, ion spray voltage 5500 V, accumulation time 0.25 s, declustering potential 60-100 V and collision energy 7 V. An automated information dependent acquisition (IDA) approach was chosen for data collection. The TOF-MS data were acquired with high resolution mass measurement (HRMS) and isotopic resolution. Ion intensity signal less than 1000 cps (count per second) was not considered for evaluation. The resulting ion intensity matrices were normalized to be expressed as a percentage relative to the most intense ion in the spectrum (taken as 100%). Using the X500R’s high-resolution mass spectrum TOF-MSIDA, with a single sample injection, TOFMS and TOF-MS/MS mass spectra were obtained. Using primary high-resolution mass numbers and the MS/MS database for comparison (e.g CAS registry/SciFinder, ChemSpider, Dictionary of Natural Products), published database searches and structural verification were performed for identification of the compounds.

### 2.6 Statistical analysis

All the assays were repeated five times and results were shown as mean ± standard deviation. Linear regression analysis was used to calculate the IC_50_ values. Pearson’s correlation coefficient was calculated and statistical significance was determined with one way ANOVA test. A statistical significance of p < 0.05 was considered to be significant. The principal component analysis (PCA) was used to show the variation of multivariate data set in terms of components. This study includes ion intensity signals of components present in root, stem, leaf, and flower. The visual presentation PCA has been done through R software. In R software biplot function and library(ggfortify) has been used to plot PCA. Heatmaps were created using the statistical software Python. Ion intensity data peak were normalized and an average value of five replicate was used for construction of heatmap.

## 3 Result

### 3.1 Compound identification

**Figure 1:**
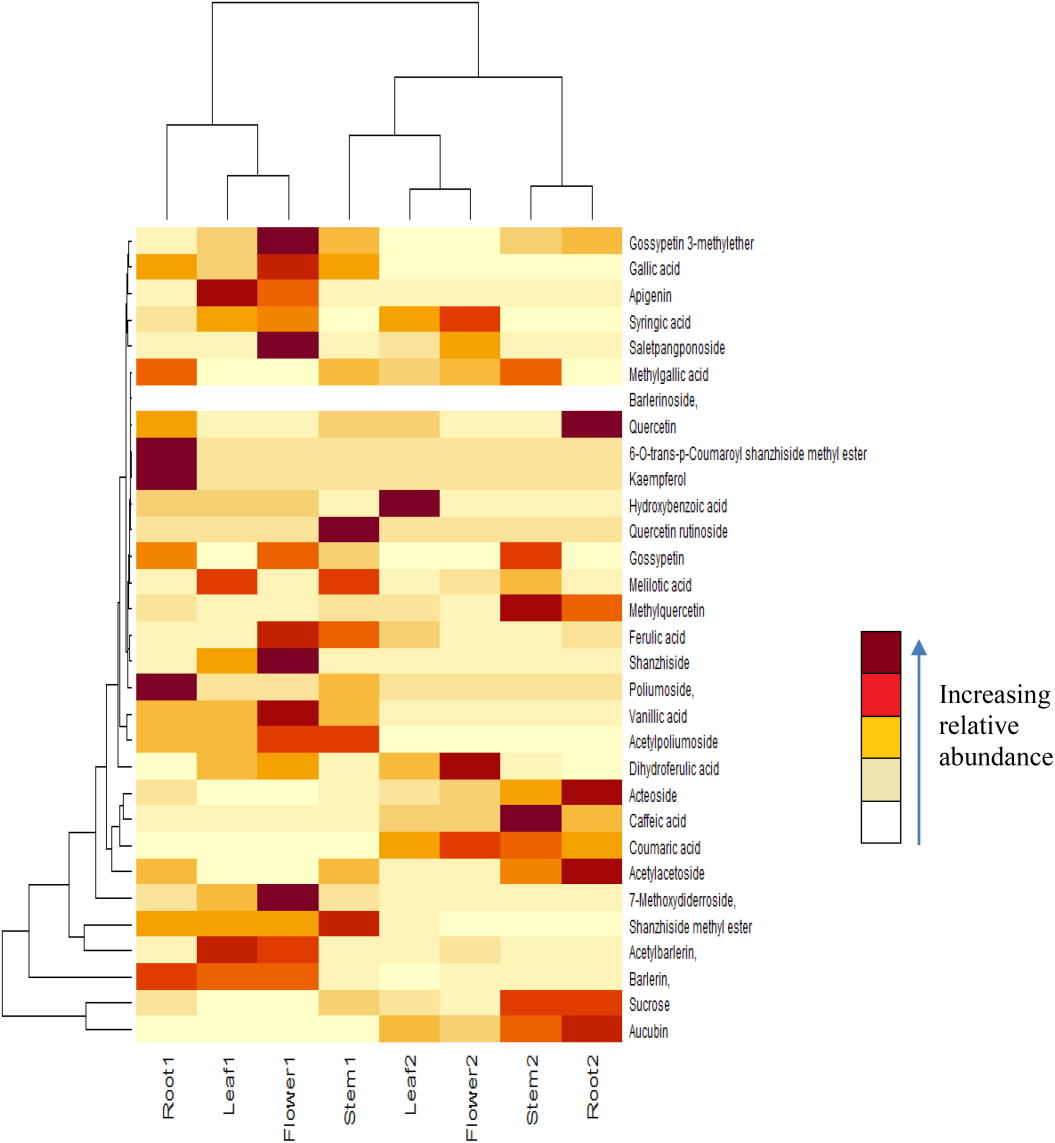
Heat map of compounds identified in different parts of *B prionitis* and *B cristata*. Root 1, Leaf 1, Flower 1 and Stem 1 are parts of *B prionitis*, whereas Leaf 2, Stem 2, Flower 2 and Root 2 are parts of *B cristata*.

ESI-MS in positive ion mode was applied for identification of active compounds from the aerial and underground parts of *Barleria* species. A total of 102 ions were detected from the mass spectra of leaf, root, stem and inflorescence of the plants. We were able to identify 58 ions, belonging to phenolic and iridoid group. A clear difference was observed in the chemical profile of both the plants as well as within different parts of the plant, showing variation in distribution and abundance of the compounds (**Table 1**; **Figure 1**). Abundant sodium and potassium adducts prevailed in the positive spectra. Adducts were determined by their mass difference from their protonated ions. Compounds were identified based on their MS1 and MS2 signals as well as isotopic distribution.

**Table 1:** Compounds identified in different parts of *B. prionitis* and *B. cristata* and their relative abundance in percentage.

	Mass peak (m/z)	Compounds identified		MS/MS Diagnostic ions	Relative Abundance (%)							
					*B. prionitis*				*B. cristata*			
		Name	Ions selected		BYL	BYR	BYS	BYF	BWL	BWR	BWS	BWF
1.	167.0682	Melilotic acid	[M+H] ^+^	149	2.2	–	2.2	–	–	–	1.2	0.3
2.	195.0674	Ferulic acid	[M+H] ^+^	177	–	–	2.5	3.2	0.9	0.5	–	–
3.	191.0300	Vanillic acid	[M+Na]^+^	173	5.1	5.0	6.0	15.3	–	–	–	–
4.	203.0843	Coumaric acid	[M+K]^+^	186	–	–	–	–	10.2	11.6	15.3	18.7
5.	208.9808	Gallic acid	[M+K]^+^	191	2.4	2.9	3.2	5.8	–	–	–	–
6.	219.0240	Caffeic acid	[M+K]^+^	163	0.5	–	0.4	0.3	3.5	4.4	12.5	3.4
7.	221.0856	Syringic acid	[M+Na]^+^	203	3.5	1.3	–	3.5	3.3	–	–	5.1
8.	223.0966	Methylgallic acid	[M+K]^+^	–	–	0.5	0.3	–	0.2	–	0.5	0.3
9.	235.1651	Dihydroferulic acid	[M+K]^+^	217	7.1	2.8	3.5	8.1	6.9	2.8	3.2	15.5
10.	293.0643	Apigenin	[M+Na]^+^	275, 199	5.2	–	–	3.2	–	–	–	–
11.	315.1582	Hydroxy benzoic acid	[2M+K]+		0.3	0.2	–	0.3	1.5	–	–	–
12.	301.1396	Gossypetin	[M-OH+H]^+^	–	0.02	1.0	0.5	1.2	–	–	1.5	–
13.	325.0939	Quercetin	[M+Na]^+^	–	–	0.4	0.2	–	0.2	1.3	–	–
14.	339.0626	Methylquercetin	[M+Na]^+^	–	–	0.5	0.4	–	0.5	1.9	3.1	–
15.	371.1324	Gossypetin 3methylether	[M+K]^+^	340, 353	2.1	1.1	2.5	5.4	1.1	2.5	2.2	0.7
16.	381.0821	Sucrose	[M+K]^+^	181	3.2	18.5	35.7	4.3	24.5	100	98.8	6.6
17.	385.0883	Aucubin	[M+K]^+^		–	–	–	–	27.4	69.9	52.7	21.3
18.	431.1574	Shanzhiside	[M+K]^+^		1.3	–	–	2.7	–	–	–	–
19.	445.1135	Shanzhiside methylester	[M+K]^+^	283, 265, 247, 233, 229, 215, 187,159,	32.3	30.5	53.5	29.5	7.5	5.4	4.9	4.2
20.	471.1503	Barlerin	[M+Na]^+^	411, 249, 231, 217, 203, 191, 177, 159, 131	83.3	100	5.5	90.3	1.2	2.7	2.1	7.5
21.	517.1443	7-Methoxydiderroside	[M+K]^+^	457, 355	11.5	1.1	1.5	40	–	–	–	–
22.	529.1350	Acetylbarlerin	[M+K]^+^	487, 427, 265	67.9	3.5	4.3	60.3	1.2	1.5	0.04	4.5
23.	543.1370	Caffeic acid derivative		163	0.2	5.4	4.1	0.3	3.4	20.5	18.7	1.2
24.	555.1131	Kaempferol	[M+K]^+^	537	–	0.8	–	–	–	–	–	–
25.	591.1534	6-O-trans-p-Coumaroyl shanzhiside methyl ester	[M+Na]^+^	–	–	1.5	–	–	–	–	–	–
26.	593.1634	Saletpangponoside	[M+K]^+^	–	–	–	–	7.2	0.5	–	–	3.4
27.	611.3609	Quercetin rutinoside		285, 325	–	–	1.5	–	–	–	–	–
28.	630.2427	Caffeic acid glycoside		612, 449	8.4	0.1	0.1	62.5	–	–	–	2.1
29.	663.4591	Acetoside	[M+K]^+^	163, 517,495, 365, 383, 177	0.03	2.2	1.0	0.02	3.1	15.6	7.6	3.5
30.	705.1915	Acetylacetoside	[M+K]^+^	663	–	10.5	10.8	–	0.9	30.6	18.5	2.2
31.	775.2332	Caffeic acid derivative		163	–	–	–	–	–	5.2	1.8	–
32.	793.2606	Poliumoside	[M+Na]^+^	163, 630	–	4.5	1.2	0.04	–	–	–	–
33.	893.2803	Acetylpoliumoside	[M+Na]^+^	793	4.5	4.0	8.3	9.2	–	–	–	–
34.	955.2951	Barlerinoside	[M+Na]^+^	–	–	–	–	–	–	–	–	–

B.prionitis: BYL-leaf extract, BYR-root extract, BYS=-stem extract, BYF- inflorescence extract.

B.cristata: BWL-leaf extract, BWR-root extract, BWS=-stem extract, BWF- inflorescence extract.

### 3.2 Identification of iridoid glycosides

Major iridoid glycosides detected in both the plants were shanzhiside methyl ester, barlerin and acetylbarlerin. Along with them Shanzhiside and 7-methoxydiderroside were observed in *B. prionitis* and aucubin in *B. cristata* (**Table 1**). Their identity was confirmed by comparing their exact mass (m/z) and fragmentation pattern (wherever applicable) from the databases and study of their isotopic distribution. Shanzhiside methyl ester, barlerin and acetylbarlerin were the most common compound present in the extracts, however their abundance was high in *B. prionitis* (**Figure 1**).

**Figure 2:**
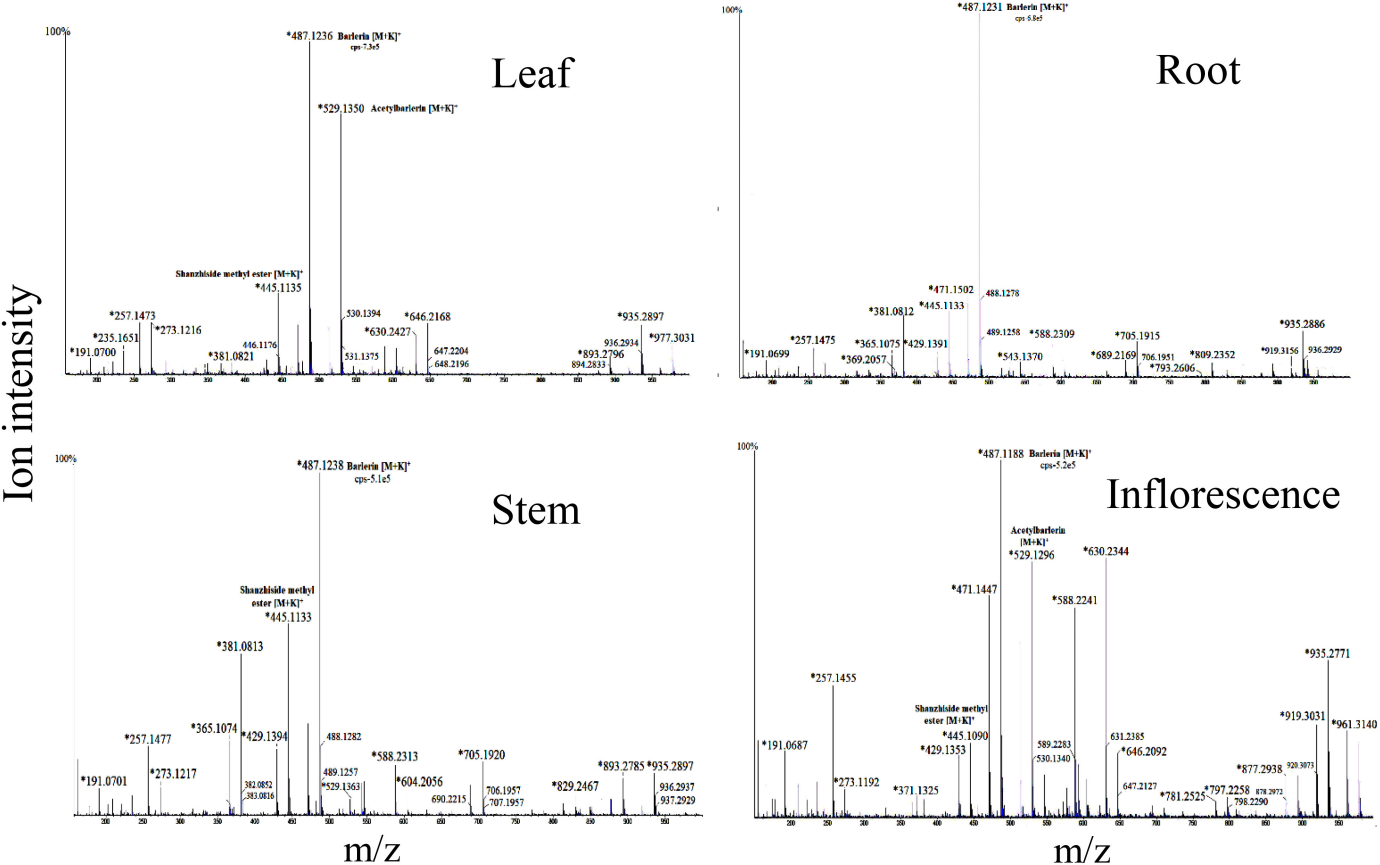
Mass spectra of root, leaf, stem and inflorescence of *Barleria prionitis* showing MS1 ions in positive ion mode. Ions marked with asterisk (*) symbol are the parent ion and without the symbol are their isotopes.

**Figure 3:**
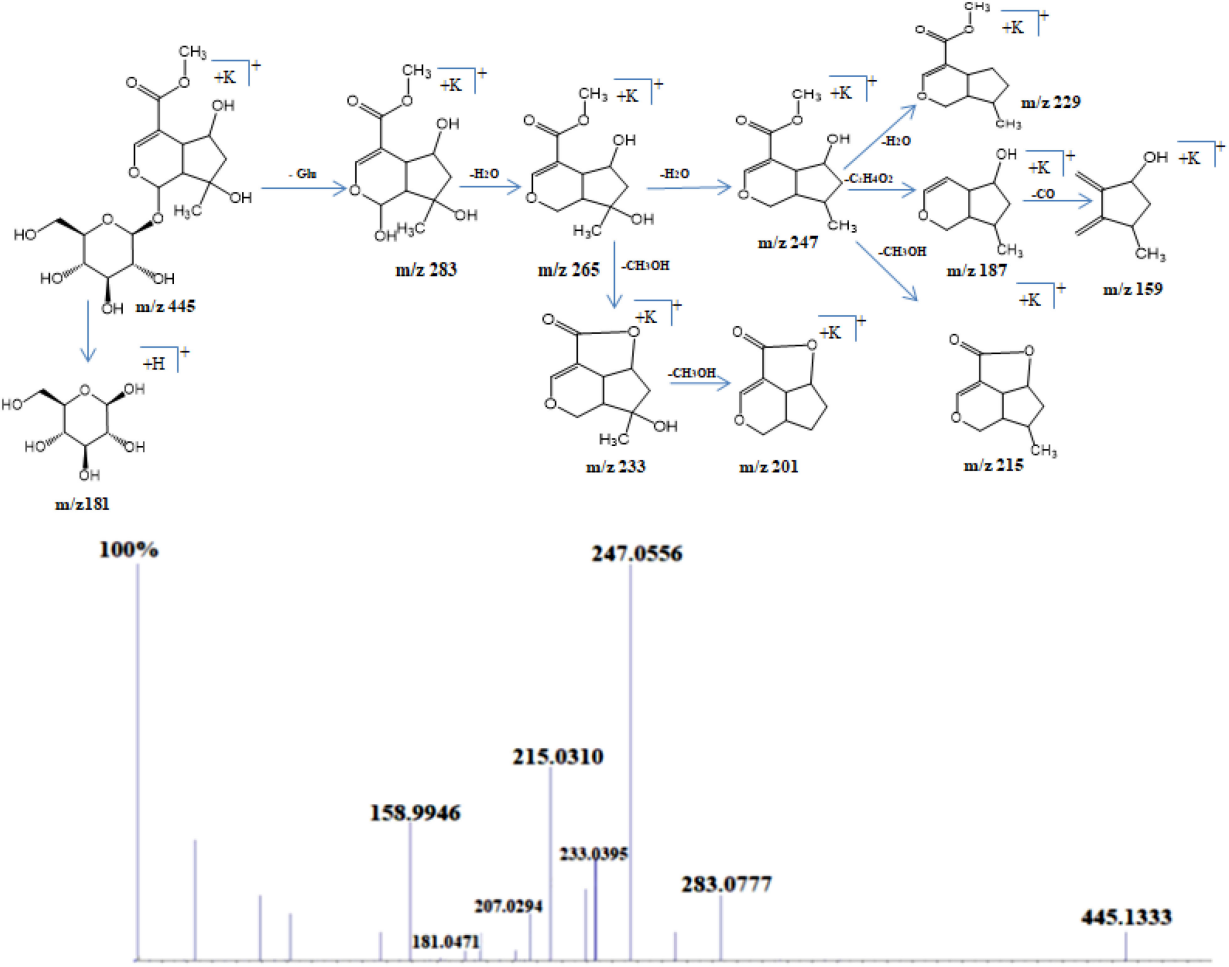
Schematic representation of fragmentation pattern of Shanzhiside methylester generated by MS/MS analysis.

**Figure 4:**
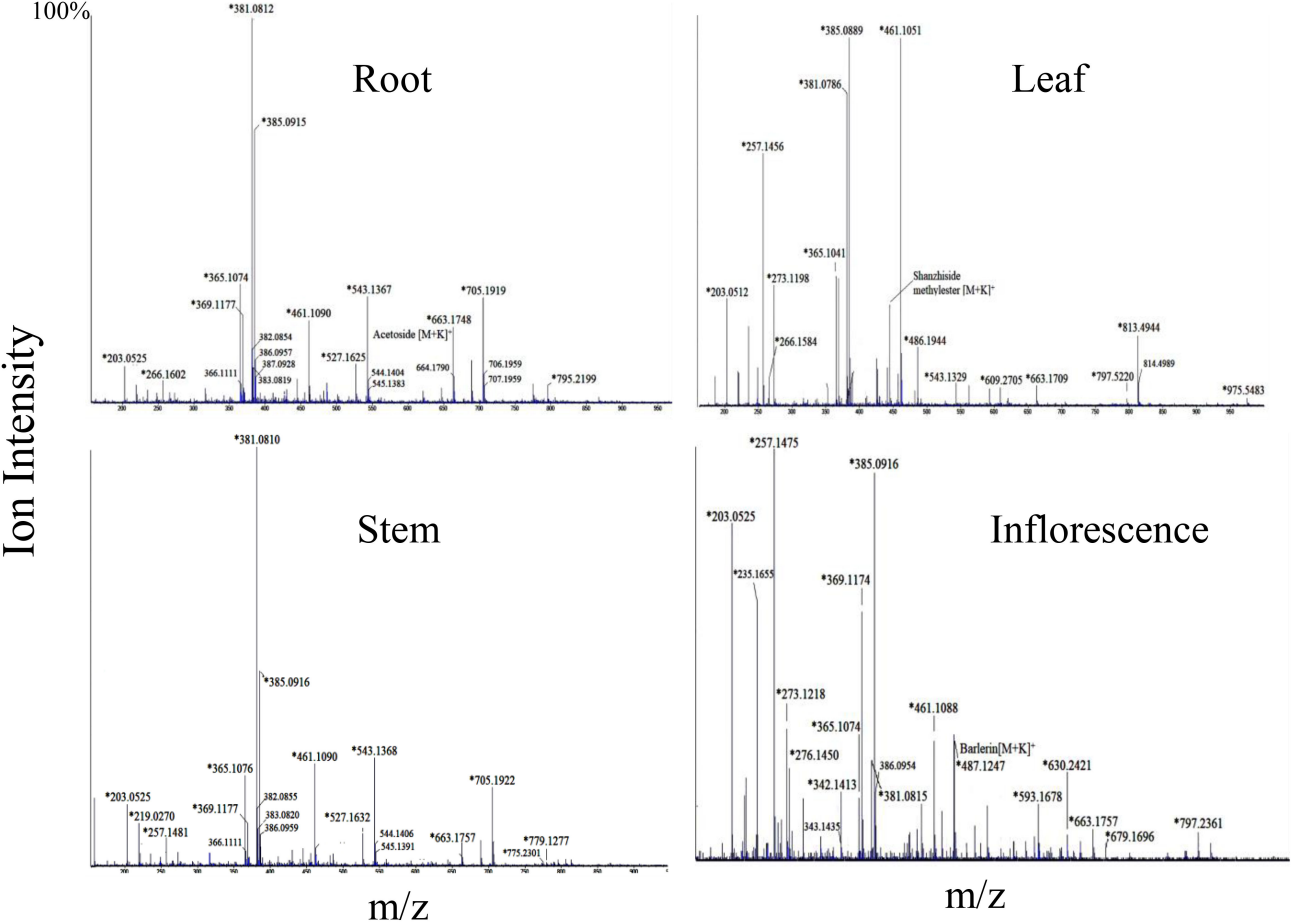
Mass spectra of root, leaf, stem and inflorescence of *Barleria cristata* showing MS1 ions in positive ion mode. Ions marked with asterisk (*) symbol are the parent ion and without the symbol are their isotopes.

Shanzhiside methyl ester was observed as sodium adduct [M+Na]^+^ with m/z 429, potassium adduct with m/z 445 and potassium dimer [2M+K]^+^ with m/z 851. Isotopes of this compound observed at m/z 446 and 447 showed a normal distribution pattern of 10:3:1 (**Figure 2**). Product ion spectra for both sodium and potassium adduct were generated. Since both ions were present in abundant amount, hence were selected by the system for MS/MS analysis (**Figure 3**). Fragmentation pattern of potassium adduct of Shanzhiside methyl ester at m/z 445 is explained in **Figure 4**, where we can clearly observe the removal of sugar moiety (162 Da) from the iridoid part generating ion at m/z 283. The most abundant ion at m/z 247 is formed by loss of two water molecules from the iridoid part. Loss of one more water molecule gave rise to signal at m/z 229, loss of C_2_H_4_O_2_ gave rise to ion at m/z 187 and loss of CO generates ion at m/z 159. A hydroxyl group is linked at C-6 position in shanzhiside methyl ester, so it easily losses a methanol molecule to form a lactone with the methylacetate (COOCH_3_) group at the C-4 position, generating product ions at m/z 233 and 215.

**Figure 5:**
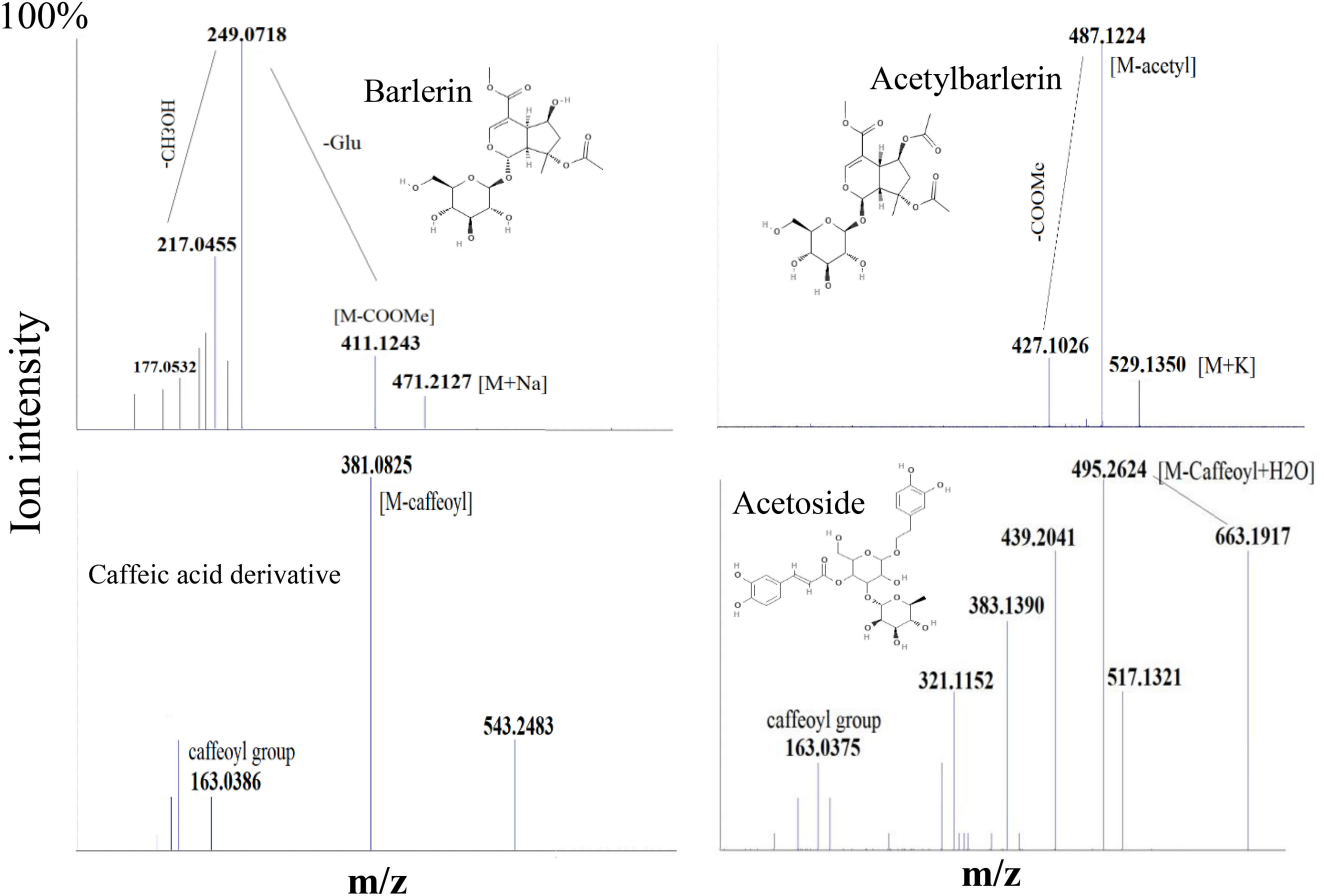
MS/MS spectra of some selected ions, showing fragmentation pattern of Barlerin, Acetylbarlerin, Caffeic acid derivative and Acetoside.

Barlerin was observed as sodium adduct [M+Na]^+^ at m/z 471, potassium adduct at m/z 487, sodium dimer [2M+K]^+^ at m/z 919 and potassium dimer [2M+K]^+^ at m/z 935, with a normal isotopic distribution pattern. MS/MS analysis of barlerin at m/z 471 [M+Na]^+^ gave fragment ion at m/z 411 formed by cleavage of methylacetate (COOCH_3_) group from the parent ion and at m/z 249 formed by removal of glucose group. Loss of water molecule gives a peak at m/z 231, whereas loss of methanol (32 Da) give rise to a peak at m/z 217. In barlerin also, a hydroxyl group is linked at C-6 position, so it losses a methanol molecule to form a lactone with the COOCH_3_ group at the C-4 position. Loss of CO group (28 Da) from 231 ion give rise to peak at m/z 203. Replacement of sodium ion with hydrogen from 231 followed by loss of water gives peak at 191. Loss of water from 217 and replacement of sodium ion with hydrogen, yields a peak at m/z 177 Da. Further loss of water followed by removal of CO, yields a peak at m/z 159 and 131 respectively (**Figure 5**).

Acetylbarlerin was observed as sodium adduct [M+Na]^+^ at m/z 513 and potassium adduct at m/z 529. Fragmentation pattern of potassium adduct of Acetylbarlerin at m/z 529 [M+K]*^+^*, shows formation of barlerin at m/z 487 [M+K]^+^ created by removal of acetyl group. Ion at m/z 427 is formed by loss of COOCH_3_ group and ion at m/z 265 by loss of glucose molecule (**Figure 5**).

### 3.3 Identification of phenolic compounds

Major groups of phenolic compounds identified were phenolic acids, flavonoids and phenylethanoid glycosides. Phenolic acids observed were melilotic acid, caffeic acid, ferulic acid, vanillic acid, coumaric acid, gallic acid, syringic acid, methylgallic acid and dihydroferulic acid (**Table 1**). Their identity was confirmed by HRMS analysis and MS/MS data comparison. MS2 signals indicating loss of water molecule (18Da) was the most important diagnostic ion generated from the parent ion peak; for example caffeic acid with m/z 181 [M+H]^+^ gave MS2 signal at 163 [M-H_2_O]^+^. Ferulic acid with m/z 195 [M+H]^+^ gave characteristic fragment ion peak at m/z 177, indicating loss of water molecule.

Flavonoids detected in *Barleria* were low in abundance (**Table 1**). Apigenin was detected by generation of fragment ion at m/z 275 formed by loss of water molecule from parent ion at m/z 293 [M+Na]^+^ and ion at m/z 199 formed by elimination of C_6_H_5_OH (phenol) group from the parent ion. MS2 signal for Gossypetin 3-methylether was also observed due to its higher abundance (heat map). Fragment with m/z 340 was generated by loss of water molecule and 353 by loss of CH_3_OH group from the parent ion. A flavonoid glycoside, quercetin rutinoside was identified by its diagnostic fragment ion 285 and 325 formed by breakage of sugar group from the parent ion.

Acetoside/Verbascoside, a caffeoyl phenylethanoid glycoside in which the phenylpropanoid caffeic acid and the phenylethanoid hydroxytyrosol form an ester and an ether bond respectively to the rhamnose part of a disaccharide, was detected in all parts of the plant, however it’s abundance was high in *B. cristata* (Heat map). It was observed as sodium adduct [M+Na]^+^ at m/z 647 and potassium adduct [M+K]^+^ at m/z 663. Fragment ions of acetoside at m/z 663 [M+K]^+^, generated characteristic ion of caffeoyl group (m/z 163). Ion at m/z 517 was produced by loss of caffeoyl group and addition of water molecule to the parent ion [M-caffeoyl+H_2_O+K]^+^. Ion at m/z 495 was generated by loss of rahmnose group from the parent ion with replacement of potassium ion with water molecule as adduct [M-Rha+H_2_O-K]^+^. Cleavage of hydroxyltyrosol group was observed, as indicated by appearance of ion at m/z 177 [hydroxyltyrosol+K]^+^. Dissacharide sugar group was cleaved from the parent molecule to generate fragment at m/z 365 [sugar+K]^+^ and addition of water generated fragment at m/z 383 (**Figure 5**).

Acetylacetoside was observed in significant amount in root and stem of the plant forming sodium and potassium ions at m/z 689 and 705 respectively. It was identified by formation of acetoside ion after removal of acetyl group. Poliumoside, a caffeoylated phenylpropanoid glycoside was also detected in significant amount in root and stem of *B.prionitis*, generating ions at m/z 793 [M+Na]^+^ and 809 [M+K]^+^. It was identified by its characteristic fragment ion m/z 630 formed by removal of caffeoyl group (163) from the parent ion (m/z 793). Acetylpoliumoside was detected by presence of its ions at m/z 893 [M+Na]^+^ and 914 [M+K]^+^ formation of poliumoside ion by loss of acetylgroup (**Table 1**).

Two ion peaks, at m/z 527 and 543 were identified as caffeic acid derivative because they generated fragment ion peak at m/z 163, indicating loss of caffeoyl group. Both signals were assumed to be generated by a single compound forming adduct with sodium and potassium ion. Similarly, ion with m/z 461, 775 and 795 observed in *B.cristata* also generated caffeoyl group fragment at m/z 163. MS/MS spectra of ions at m/z 257 and 273 were quite similar showing loss of hydroxyl group (18Da), indicating them to be a phenolic compound forming sodium and potassium adduct. Similarly, ions with m/z 604 and 630 observed in *B.prionitis* were also observed as signal of a similar compound indicating loss of water molecule (18Da) and glycosyl group; showing their probability to be a phenolic glycoside (**Figures 2**, **4**).

### 3.4 Antioxidant and antibacterial activity

In our experiments, a strong antioxidant activity was observed in all parts of the plant by DPPH and FRAP assay, demonstrating free radical scavenging activity of the compounds (**Table 2**). A comparative analysis indicates highest activity in root and leaf of *B.cristata*. In *B.prionitis* highest activity was shown by inflorescence, followed by root. Antibacterial activity was shown in all parts of the plant, with root being the most effective one in both the species (**Table 2**). All parts were effective against *S. aureus-aureus*, with highest values shown by root extract. *B.cristata*, leaf, root and inflorescence were effective against most of the tested bacteria, whereas *B.prionitis* root extract was only effective against *P.aeruginosa, B.subtilis* and *E.coli*.

**Table 2:** Antioxidant and antibacterial activity of extracts obtained from different parts of *B.prionitis* and *B.cristata*.

	Antioxidant activity		Antibacterial activity (extracts 10 mg/ml)- zone of inhibition (cm)				
	FRAP assay (mgAA/g)	DPPH assay IC50 μg/mL	Staphylococcus aureus-aureus	Klebsiella pneumoniae	Psuedomonas aeruginosa	Bacillus subtilis	Escherichia coli
**BYL**	112.71 ± 5.67	62.2 ± 4.67	1.2 ± 0.3	–	–	–	–
**BYR**	141.92 ± 10.34	48.3 ± 3.45	2.0 ± 0.5	–	1.2 ± 0.1	1.3 ± 0.2	1.2 ± 0.2
**BYS**	104.85 ± 8.23	77.6 ± 6.35	1.1 ± 0.2	–	–	–	–
**BYF**	156.76 ± 12.24	38.5 ± 3.56	1.4 ± 0.3	–	–	–	–
**BWL**	174.69 ± 13.45	29.3 ± 4.53	1.7 ± 0.2	1.4 ± 0.2	1.5 ± 0.3	1.4 ± 0.3	1.2 ± 0.1
**BWR**	208.74 ± 14.36	18.4 ± 3,21	2.2 ± 0.4	1.2 ± 0.1	1.7 ± 0.4	1.5 ± 0.3	1.4 ± 0.2
**BWS**	143.04 ± 12.66	47.2 ± 6.57	1.6 ± 0.1				
**BWF**	128.17 ± 12.48	65.5 ± 6.55	1.7 ± 0.1	1.5 ± 0.4	1.4 ± 0.2		1.3 ± 0.1
**Ascorbic acid**		9.2 ± 2.38					
**Streptomycin (1mg/ml)**			3.1 ± 0.6	2.7 ± 0.5	2.3 ± 0.5	3.2 ± 0.6	2.6 ± 0.3

Values are expressed as mean ± SE (n = 5).

### 3.5 Correlation analysis

**Figure 6:**
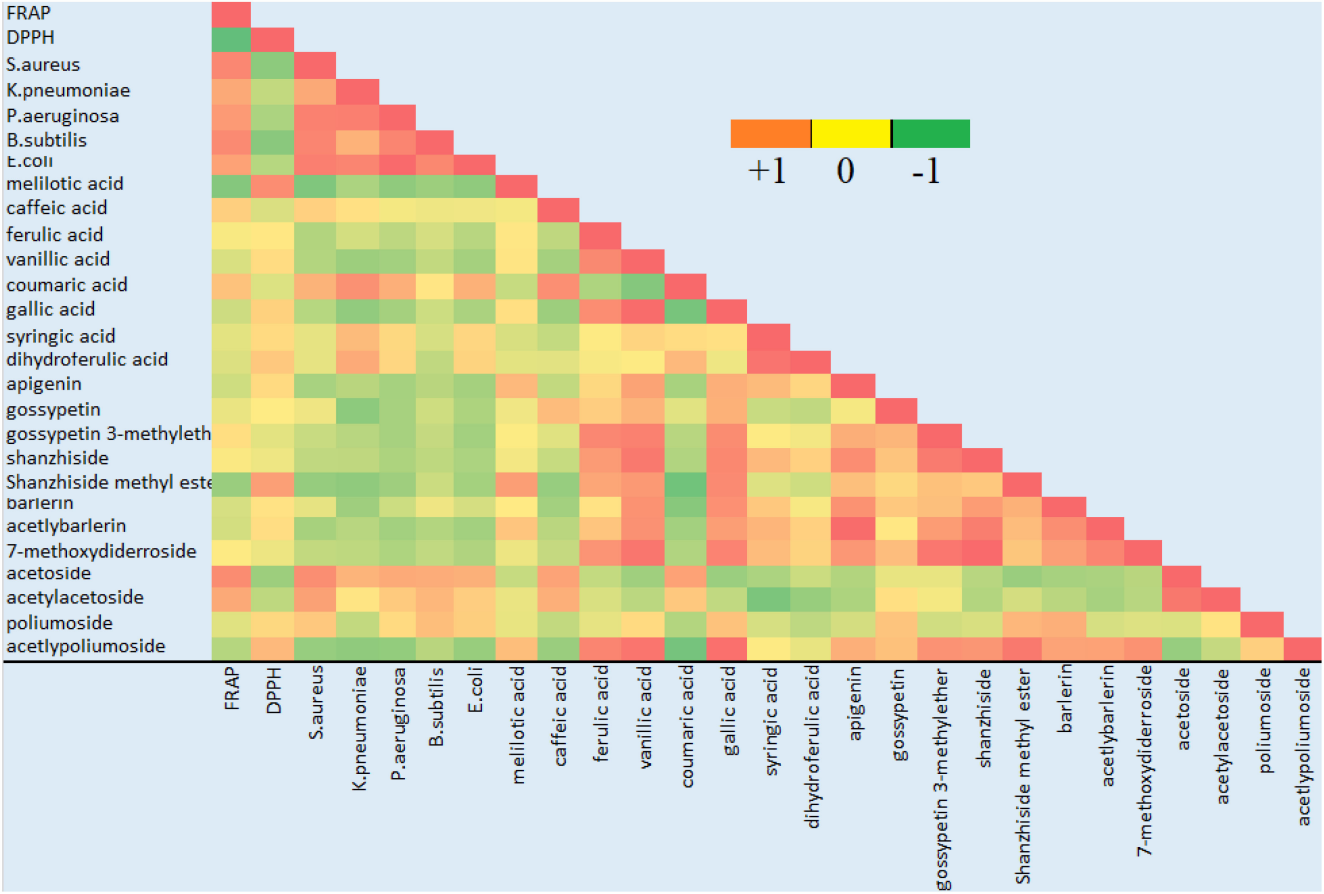
Correlation analysis of the identified compounds with antioxidant and antibacterial activity. Green color indicates negative correlation, red color indicates positive correlation and shades of yellow show no correlation.

Correlation study was conducted to identity the contribution of various compounds in antioxidant and antibacterial activity. The correlation coefficients between the ion intensity peaks of the compounds and the bioactivity results, including FRAP assay, IC50 value for DPPH assay and antibacterial activity against *S. aureus-aureus* was calculated against 95% confidence level (**Figure 6**). As we can see from the table that most of the phenolic acid and flavonoids showed a positive correlation with the antioxidant activity. Phenylethanoid glycoside acetoside, poliumoside and acetylpoliumoside showed a positive correlation with antioxidant activity. Some of the phenolic compounds were positively correlated with antibacterial activity, among which caffeic acid and coumaric acid were the most important one. Among flavonoids gossypetin was positively correlated, phenylethanoid glycoside including acetoside, acetylacetoside and poliumoside were also positively correlated and among iridoid glycoside barlerin was slightly positively correlated with the antibacterial activity.

### 3.6 Principal component analysis

**Figure 7:**
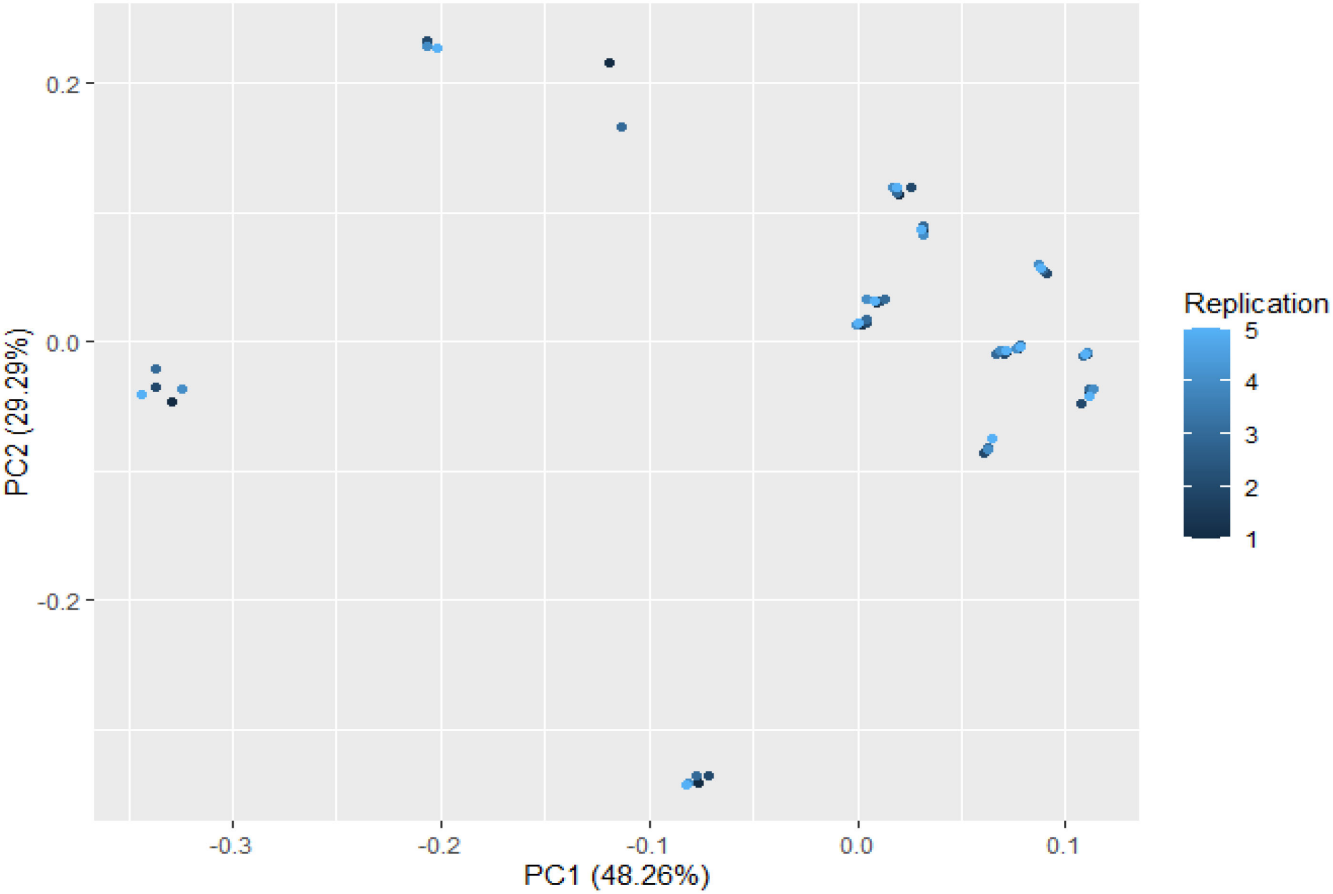
Scatter plot of first two principal components PC1 and PC2 with the replication of 1 to 5 presented. This plot shows variability in the distribution pattern of metabolites.

**Figure 8:**
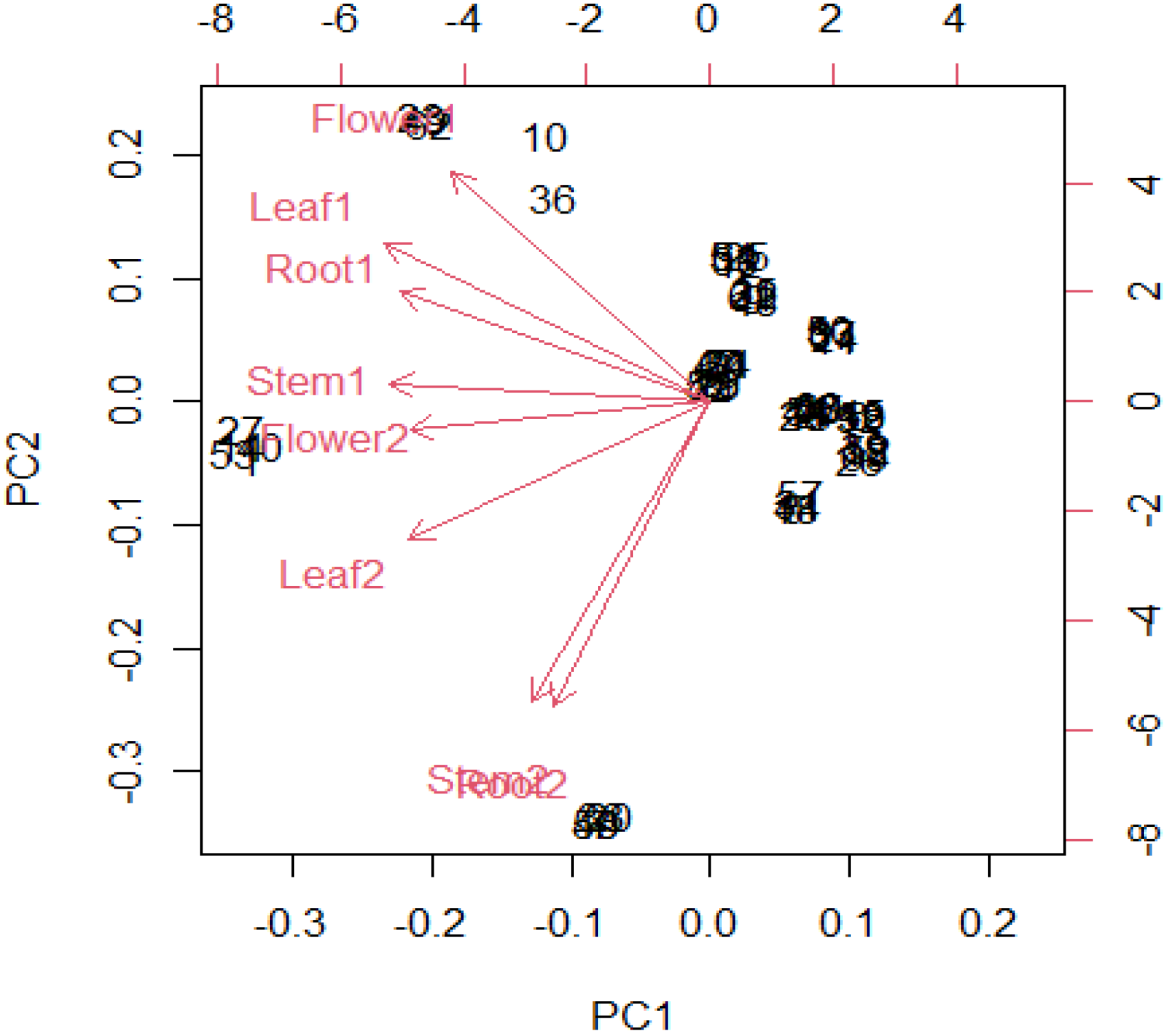
In R software biplot function has been used to plot PCA. The given biplot represents the first principal component which combine (flower, leaf) and little part of variation in (stem, root) in second principal component.

**Figure 9:**
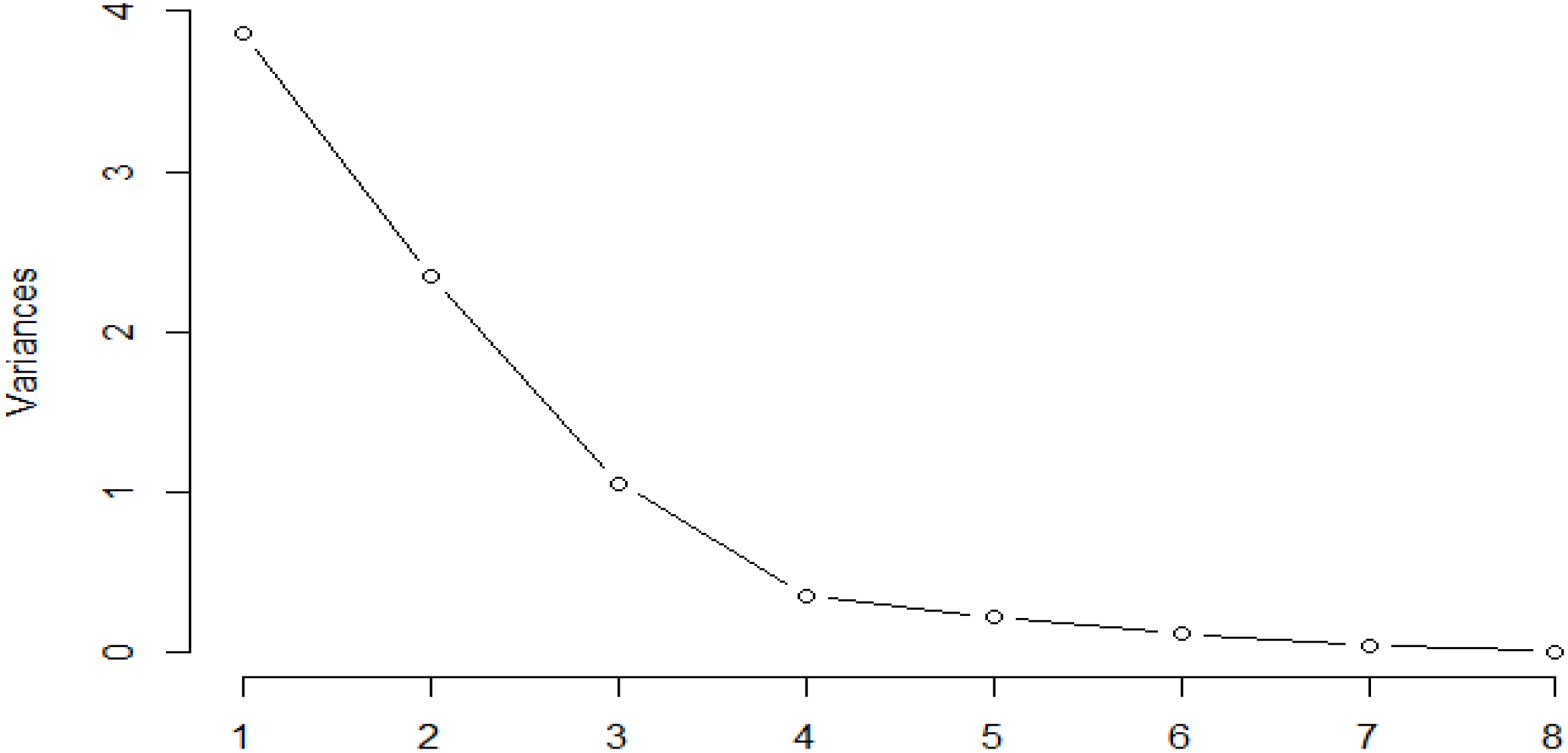
Screen plot represents decreasing pattern of cumulative contribution of variation for eight principal components.

Thirteen ion peaks were ubiquitously distributed in all parts of both the plants, therefore they were selected as marker signals for PCA analysis, among which shanzhiside methylester, barlerin, acetylbarlerin and acetoside were the identified compounds. The total percentage of variation of data set was explained using four principal components (PC1, PC2, PC3 and PC4). The results showed that PC1 explains 48.26 percent, PC2 explains 29.29 percent, PC3 13.21 percent and PC4 explains 4.42 percent variation of dataset (**Table 3**). PC1 explains the maximum variation as compared to the other PCA in whole dataset. Scatter plot represents the variation of first two principal components PC1 and PC2 within the replicate 1 to 5 (**Figure 7**), demonstrating non-linear pattern of data which does not show normality. The given biplot in **Figure 8** represents the first principal component which combines flower, leaf and some part of variation in stem and root with second principal component. The Screen plot represents a decreasing pattern of cumulative contribution of variation for eight principal components (**Figure 9**).

**Table 3:** Variance explained by the PC1-PC8 with standard deviation and cumulative proportion.

	PC1	PC2	PC3	PC4	PC5	PC6	PC7	PC8
**Standard Deviation**	1.9648	1.5308	1.0282	0.5944	0.4652	0.3441	0.2125	0.0752
**Proportion of variance**	0.4826	0.2929	0.1321	0.0442	0.0271	0.0148	0.0057	0.0007
**Cumulative proportion**	0.4826	0.7755	0.9076	0.9518	0.9786	0.9937	0.9992	1.0000

## 4 Discussion

A rapid metabolomic approach was used in the present research work for chemical profiling of active compounds in *B.prionitis* and *B.cristata*. X500R QTOF system uses information dependent acquisition (IDA) method to collect high resolution accurate mass data (HRMS) on both the parent and their fragment ions. IDA performs a non- targeted screen of MS1 fingerprint and separates MS1 signals into small ion currents, further selecting the most abundant ion current from the TOF MS total ion chromatogram to perform MS/MS fragmentation (Whitman and Lynch, 2019). The whole process was accomplished in a single run, thereby overcoming separation requirement of the extract. Compounds were identified on the basis of their HRMS analysis, fragmentation pattern and isotopic distribution, as reported in literature (Heffels et al., 2017; Kite, 2020). Most of the natural compounds are made up of carbon, hydrogen, oxygen and nitrogen. Their protonated molecules have similar isotope patterns and the intensities of their isotopic ions are always in descending order: M > M+1 > M+2, following the ratio 10:3:1 (Zhang et al., 2012). Many of the detected compound such as shanzhiside methylester and barlerin showed a similar isotopic abundance, which helped us in further identification.

Iridoid glycosides and phenolic compounds have been reported from aerial parts of *Barleria* species (Gangaram et al., 2022), however as shown in our study, they are distributed in all parts of the plant, including leaf, root, stem and flower (**Figure 2**). The study also helped us to analyse the distribution pattern and abundance of these compounds in both the plants. Principal component analysis (PCA), conducted according to the methods described in literature (Abdi and Williams, 2010; Bro and Smilde, 2014; Lever et al., 2017), showed variation in the distributed pattern of identified metabolites. We can observe that *B. prionitis* had high relative abundance of iridoid glycosides, particularly shanzhiside methy ester, barlerin and acetylbarlerin, whereas *B. cristata* showed higher relative abundance of acetoside. These metabolites are active compounds with broad spectrum of biological activities. Extracts enriched with iridoid glycosides have shown glutathione S-transferase inhibitory activity, acetylcholinesterase inhibitory activity, free radical scavenging, antimicrobial, anti-inflammatory, immunomodulatory and gastroprotective activities (Kosmulalage et al., 2007; Ata et al., 2009; Ghule and Yeole, 2012; Jaiswal et al., 2014). Shanzhiside methyl ester and barlerin enriched fraction have shown to modulate specific and non-specific immune response in *in vivo* studies (Ghule and Yeole, 2012). Shanzhiside methyl ester have shown to reduce neuropathic pain by stimulating spinal microglial β-endorphin expression (Zhang et al., 2018), it also possesses anti-inflammatory properties and have shown to provide protection against depression by inhibiting inflammation (Sun et al., 2022). Iridoid glycoside 6-O-transp-coumaroyl-8-*O*-acetylshanzhiside methyl ester and its *cis* isomer have shown activity against respiratory syncytial virus (Chen et al., 1998). Similarly, phenylethanoid glycosides, a group of phenolic compound made up of phenylethyl alcohol, caffeic acid and glycosyl groups (Leea et al., 2016) shown in our results have reported biological activity. Barlerinoside, a phenylethanoid glycoside, isolated from aerial parts of *Barleria* sps. have shown to possess glutathaione S transferase and acetylcholinesterase inhibitory activity (Ata et al., 2009). Verbascoside/acetoside, a phenylpropanoid glycoside identified in *Barleria* species is known for its antioxidant, analgesic, anticancer, anti-inflammatory and photoprotective activity (Vertuani et al., 2011). Identification of these active metabolites in different parts of both the species will help in exploiting full pharmacological potential of the plant.

Some studies have demonstrated antioxidant and antibacterial activity of *Barleria* sps. (Amoo et al., 2009; Sunil et al., 2010; Chavan et al., 2014), however our study shows a comparative analysis, proving *B.cristata* to be more effective against tested bacterial species. As we can see that most of the phenolic acid and flavonoids showed a positive correlation with the antioxidant activity. Phenolic compounds are widely distributed plant substances and have been considered as significant contributors to antioxidant activity (Bittencourt et al., 2015; Kozyraa et al., 2019; Pannakal et al., 2022; Singh, 2023). Some of the phenolic compounds were positively correlated with antibacterial activity, among which caffeic acid and coumaric acid were strongly correlated. Earlier reports also confirms their role as antimicrobial (Bittencourt et al., 2015; Merlani et al., 2019). Acetoside has an antimicrobial activity, notably against *Staphylococcus aureus* (Bazzaz et al., 2018). None of the detected iridoid glycoside showed to be a major contributor in antioxidant and antibacterial activity, except barlerin having positive correlation with the tested bacterial strain. Owing to the abundance of these compounds in the tested extract it could be concluded that either alone or synergistically they might be responsible for the biological activity.

## 5 Conclusions

Phytochemical investigation of *B. prionitis* and *B. cristata* led to the identification of iridoid glycosides and phenolic compounds in different parts of the plant. Most abundant iridoid glycosides were barlerin, acetylbarlerin and shanzhiside methylester, whereas acetoside and acetylacetoside were the important phenolic compound present in the plants. Many of the chemical compounds are reported for the first time in *Barleria* species, such as ferulic acid, caffeic acid, kaempferol, acetlyacetoside. Most of the compounds detected in root, stem and inflorescence have not been reported earlier. Chemical compounds were found to be distributed in all parts of the plant with variation in their presence and abundance. They were shown to be correlated with antioxidant and antibacterial activity.

## Data availability statement

The original contributions presented in the study are included in the article/supplementary material. Further inquiries can be directed to the corresponding author.

## Author contributions

All authors listed have made a substantial, direct, and intellectual contribution to the work, and approved it for publication.
